# Wnt Inhibitory Factor 1 Binds to and Inhibits the Activity of Sonic Hedgehog

**DOI:** 10.3390/cells10123496

**Published:** 2021-12-10

**Authors:** Krisztina Kerekes, Mária Trexler, László Bányai, László Patthy

**Affiliations:** Institute of Enzymology, Research Centre for Natural Sciences, H-1117 Budapest, Hungary; kerekes.krisztina@ttk.mta.hu (K.K.); trexler.maria@ttk.mta.hu (M.T.); banyai.laszlo@ttk.mta.hu (L.B.)

**Keywords:** cancer, cholesterol-group, hedgehog, lipid-group, palmitoyl-group, tumor suppressor, Wnt, Wnt Inhibitory Factor 1

## Abstract

The hedgehog (Hh) and Wnt pathways, crucial for the embryonic development and stem cell proliferation of Metazoa, have long been known to have similarities that argue for their common evolutionary origin. A surprising additional similarity of the two pathways came with the discovery that WIF1 proteins are involved in the regulation of both the Wnt and Hh pathways. Originally, WIF1 (Wnt Inhibitory Factor 1) was identified as a Wnt antagonist of vertebrates, but subsequent studies have shown that in *Drosophila*, the WIF1 ortholog serves primarily to control the distribution of Hh. In the present, work we have characterized the interaction of the human WIF1 protein with human sonic hedgehog (Shh) using Surface Plasmon Resonance spectroscopy and reporter assays monitoring the signaling activity of human Shh. Our studies have shown that human WIF1 protein binds human Shh with high affinity and inhibits its signaling activity efficiently. Our observation that the human WIF1 protein is a potent antagonist of human Shh suggests that the known tumor suppressor activity of WIF1 may not be ascribed only to its role as a Wnt inhibitor.

## 1. Introduction

Wnt and hedgehog (Hh) signaling play essential roles in the control of cell proliferation and development [1,2]. The abnormal activation of the Wnt or Hh signaling pathways leads to the development of various types of cancer [3,4].

Several similarities of the Hh and Wnt pathways argue for their common evolutionary origin [5,6,7]. 

Both Wnts and Hhs are lipid-modified, and the lipid-moieties of these morphogens are essential for their activities. Wnt proteins are palmitoleoylated, and this modification is indispensable for their activity [8,9]. Hh morphogens are modified by palmitoic acid at their N-terminal end and by a cholesterol molecule at their C-terminal end [10,11,12,13]. The palmitoylation of Hh morphogens is essential for their activity; inhibition of Hh palmitoylation blocks Hh signaling [14,15,16]. 

The most striking similarity of the Wnt and Hh pathways is that signaling is mediated by closely related receptors (Frizzled and Smoothened, respectively). Additional similarities of the two pathways include the use of GSK3β, CK1, and β-TrCP to regulate the proteolysis of the key transcriptional effectors of these pathways [5,6,7]. Comparative genomics studies have revealed that the main constituents of the Frizzled/Smoothened-dependent pathways evolved in early Metazoa. According to these studies, the main constituents of the Wnt and Hh pathways were present before the divergence of Parazoa and Eumetazoa (for a recent summary, see [7]). 

The activity of Wnts is regulated by a variety of secreted extracellular proteins that interfere with the formation of the Wnt-receptor complexes [17,18,19,20]. One of these proteins is Wnt Inhibitory Factor 1 (WIF1), first identified in fish, amphibia, and mammals as a protein containing a WIF domain and five EGF-like domains [21]. Hsieh et al. have shown that human WIF1 inhibits the activity of *Xenopus* Wnt8; the inhibitory activity of the WIF domain was similar to that of full-length human WIF1, indicating that Wnt-binding is mediated by this domain [21].

The importance of WIF1 as a Wnt antagonist is illustrated by the fact that transgenic mice in which the WIF1 gene was disrupted were more susceptible to spontaneous and radiation-induced osteosarcoma than wild type animals, suggesting that WIF1 may function as a tumor suppressor [22]. The tumor suppressor role of WIF1 is also supported by the observation that the silencing of WIF1 by methylation and by noncoding RNAs contributes to a large variety of tumors (for a review, see [7]). 

In 2005, two groups found that an ortholog of vertebrate WIF1s is present in *Drosophila* [23,24]. The domain architecture of the *Drosophila* shifted (Shf) protein is equivalent to those of vertebrate WIF1 proteins: it also has a WIF domain and five EGF-like domains. WIF domains orthologous with the WIF domains of WIF1 are also found in Porifera, Cnidaria, Annelida, Brachiopoda, Mollusca, Echinodermata, and Hemichordata, suggesting that this domain-type was present prior to the divergence of Parazoa and Eumetazoa [7]. The architecture of a WIF domain containing protein found in the sponge, *Oopsacas minuta*, has remarkable similarity to eumetazoan WIF1 proteins: it has a WIF domain and three EGF domains, indicating that these two domain-types have been joined in the first animals [7]. 

Surprisingly, the function of the *Drosophila* WIF1 ortholog, Shf appeared to be quite different from the Wnt inhibitory function of vertebrate WIF1 proteins. Shf appears to have no significant role in Wnt signaling in *Drosophila*, as its overexpression did not generate Wnt-related defects [23,24,25]. Instead, the Shf protein has been shown to be required for the stability and normal levels of the Hh protein and to control the diffusion of lipid-modified Hh in the extracellular matrix [23,24]. Flies carrying mutations of the *Shf* gene differed from wild type flies in as much as Hh did not accumulate normally, and the range of Hh movement and signaling was strongly reduced, suggesting that it serves as a positive modulator of Hh signaling [23,24].

Based on these studies, it has been suggested that *Drosophila* Shf serves as a positive modulator of the Hh pathway, whereas vertebrate WIF1 proteins function only to block the activity of Wnts. In order to define the regions that are responsible for the Hh and Wnt specificity of the *Drosophila* and human WIF1 proteins, Guerrero’s group [26] has analyzed the activity of chimeric WIF1 proteins. These studies have shown that the WIF domain carries the specificity for Hh or Wnt, whereas the EGF domains are crucial for interaction of the proteins with the heparan sulfate proteoglycans of the extracellular matrix. Blair et al. [27] have also used the chimeras of vertebrate WIF1 and *Drosophila* Shf to get an insight into the structural basis of the differences in their specificities. The authors have found that full Wnt inhibition required the WIF domain of vertebrate WIF1 and the heparan sulfate proteoglycan-binding EGF-like domains of either vertebrate WIF1 or Drosophila Shf. The full promotion of Hh signaling was found to require both the EGF-like domains of Shf and the WIF domains of either WIF1 or Shf. In other words, the WIF domain of the vertebrate WIF1 protein is capable of increasing the Hh promoting activity of EGF domains of Shf, raising the possibility that WIF1 might also modulate Hh signaling in vertebrates [27]. In fact, full-length vertebrate WIF1 affected the distribution and signaling of Hh in *D. melanogaster*, suggesting a possible role for WIF1 as a modulator of vertebrate Hh signaling [27].

In summary, these studies have led to the conclusion that vertebrate WIF1 serves primarily as a negative regulator of the Wnt pathway, whereas the *Drosophila* ortholog Shf serves as a positive regulator of the Hh pathway. It remained unclear, however, whether the human WIF1 protein is highly specific for human Wnts or if it also has a significant influence on the signaling activity of human Hhs.

In the present work, we have characterized the interaction of the human WIF1 protein with human sonic hedgehog (Shh) using Surface Plasmon Resonance spectroscopy and reporter assays to monitor the influence of WIF1 proteins on the signaling activity of human Shh. Our studies have shown that the human WIF1 protein binds human Shh with high affinity and inhibits its signaling activity efficiently. Our observation that the human WIF1 protein is a potent antagonist of Shh suggests that the known tumor suppressor activity of WIF1 may not be ascribed only to its role as a Wnt inhibitor. This finding cautions that the loss of WIF1 activity might also promote carcinogenesis through the aberrant activation of the Hh pathway and that the therapeutic targeting of WIF1 might have effects on both the Wnt and Hh pathways. 

## 2. Materials and Methods

### 2.1. Proteins, Cell Lines, Media and Reagents

Recombinant human WIF1 (rhWIF1) and dually lipidated human sonic hedgehog (rhShh) proteins were purchased from R&D Systems (Minneapolis, MN, USA). The Human WIF1 Antibody (Goat IgG, AF134) and Human/Mouse Sonic Hedgehog/Shh N-Terminus Antibody (Goat IgG, AF464) were from R&D Systems (Minneapolis, MN, USA). An Anti-Goat IgG (whole molecule)-Alkaline Phosphatase antibody produced in rabbit (A4187) and Anti-Rat IgG (whole molecule)–Alkaline Phosphatase antibody produced in goat were from Sigma-Aldrich (St Louis, MO, USA). The WIF domain of WIF1 protein was produced as described previously [28].

The ONE-Step™ Luciferase Assay System and the Gli Reporter-NIH3T3 cell line were purchased from BPS Bioscience (Inc., San Diego, CA, USA). An Opti-MEM Reduced Serum Medium (Gibco by Thermo Fisher Scientific, Waltham, MA, USA) with 0.5% calf serum (Merck—SigmaAldrich, KGaA, Darmstadt, Germany), 1% non-essential amino acids (Gibco by Thermo Fisher Scientific, Waltham, MA, USA), and 1% penicillin/ streptomycin was used for reporter assays. Culture media DMEM was obtained from Merck—SigmaAldrich (KGaA, Darmstadt, Germany). Luminescence was measured using an EnSpire plate reader (PerkinElmer, Inc. Waltham, MA, USA).

A Pierce™ Classic IP Kit (26146, Thermo Fisher Scientific, Waltham, MA, USA) was used for pull-down assays.

CM5 sensor chips and the reagents for protein coupling to the chips were from Biacore AB (Uppsala, Sweden). The Amersham Protran Premium nitrocellulose blotting membrane was from GE Healthcare Life Sciences (Marlborough, MA, USA). Nitro Blue tetrazolium and 5-bromo-4-chloroindol-2-yl phosphate were from Serva Electrophoresis (Heidelberg, Germany).

### 2.2. Pull-Down Assays

The rhWIF1 protein (12 pmole) was incubated with the rhShh protein at a molar ratio of 1:1 for 1h at 4 °C in TBS buffer (25 mM Tris, 150 mM NaCl; pH 7.2; final volume: 30 μL), then antibodies against rhWIF1 or rhShh were added to the appropriate mixtures at a molar ratio of 1:1.5, and the solutions (final volume: 50 μL) were further incubated for 1 h at 4 °C. The protein-protein-antibody equilibrium mixtures (50 μL) were added to microcentrifuge columns filled with 45 μL Pierce Protein A/G Plus Agarose, 100 μL of the TBS buffer was added, and the columns were gently shaken overnight at 4 °C. The microcentrifuge columns were centrifuged, and the A/G Plus Agarose was washed with 4 × 200 μL TBS buffer and the bound proteins were eluted with 50 μL elution buffer (pH 2.8), according to the manufacturer’s instructions. The samples were analyzed by Western blotting using antibodies specific for the WIF1 or Shh proteins. 

### 2.3. Surface Plasmon Resonance Analyses

SPR measurements were performed on a BIACORE X (GE Healthcare, Stockholm, Sweden) instrument. The proteins to be immobilized were dissolved in a 50 mM sodium acetate buffer, pH 4.5, and solutions were injected with a 5 µL/min flow rate on a CM5 sensor chip activated by the amine coupling method, according to the manufacturer’s instructions. For interaction measurements, 90 µL aliquots of protein solutions were injected over the sensor chips with a 20 µL/min flow rate. Binding and washes were performed in 20 mM Tris, 150 mM NaCl, 5 mM EDTA, 0.005% Tween 20, 100 μM CHAPS, pH 7.4 buffer. After each cycle, the chips were regenerated by injection of 35 µL of 8 M urea, 1M NaCl, 100 mM Tris, 5 mM EDTA, and 0.005% Tween-20, pH 7.4. Control flow cells were prepared by performing the coupling reaction in the presence of coupling buffer alone. Control flow cells were used to obtain control sensorgrams showing nonspecific binding to the surface as well as refractive index changes resulting from changes in bulk properties of the solution. Control sensorgrams were subtracted from sensorgrams obtained with immobilized ligand. 

All experiments were repeated at least three times. To correct for differences between the reaction and reference surfaces, we also subtracted the average of sensorgrams obtained with blank running buffer injections. The kinetic parameters for each interaction were determined by fitting the experimental data with BIA evaluation software 4.1, and the closeness of the fits was characterized by the χ^2^ values. Only fits with χ^2^ values lower than 5% of the R_max_ were accepted. Data were fitted to a model of 1:1 Langmuir interaction. 

### 2.4. Reporter Assays

The signaling activity of human sonic hedgehog and the hedgehog antagonist activities of WIF1 and WIF domain proteins were assayed on the Gli Reporter-NIH3T3 cell line containing the firefly luciferase gene under the control of Gli responsive elements stably integrated into NIH3T3 cells. 

In the reporter assays, monitoring the signaling activity of sonic hedgehog 2.5 × 10^4^ cells/100 μL/well were incubated for ~24 h in DMEM/Q/calf serum/PS in 96-well tissue culture dishes in a CO_2_ incubator at 37 °C. When cells reached confluency, the medium was removed from the wells and 50 µL aliquots of a serial dilution of rhShh (0–245 nM) in assay medium (Opti-MEM Reduced Serum Medium with 0.5% calf serum, 1% non-essential amino acids, and 1% penicillin/streptomycin) were added to the wells. The dishes were incubated for 18 h, then the luminescence of the wells was measured using an EnSpire plate reader.

In the reporter assays, while monitoring the hedgehog antagonist activities of WIF1 and WIF domain proteins, the cells were grown to confluency, and after the removal of the culture medium, 50 µL aliquots of 0.2 nM rhShh, preincubated for 5 min with 0–50 nM of rhWIF1 protein or 0–50 nM of human WIF domain in assay medium, were added. 

In the case of control experiments, cells were grown to confluency, and after the removal of the culture medium, 50 µL aliquots of assay medium were added to the cells (unstimulated control wells). To determine the background luminescence of the wells, 50 µL aliquots of assay medium were added to cell-free control wells. 

The experiments were repeated four times, and each experiment had four parallels. 

Luciferase assay was performed using the ONE-Step™ Luciferase Assay System according to the protocol provided by the manufacturer: 50 µL of ONE-Step™ Luciferase reagent was added to each well and the plates were shaken at room temperature for 20 min. Luminescence was measured using EnSpire plate reader. The average background luminescence (cell-free control wells) was subtracted from the luminescence reading of all wells, and the average fold induction of Gli luciferase reporter expression was calculated by comparing the luminescence of stimulated and unstimulated wells. 

### 2.5. Protein Analyses

For Western blotting, samples were run on a non-reducing 16% SDS gel, and the proteins were transferred to nitrocellulose membranes. The membranes were blocked for 1 h at room temperature in 10 mM Tris/HCl, 150 mM NaCl, and 0.05% Tween-20, pH 7.5 (TBST) supplemented with 5% non-fat dry milk. The blots were probed with a primary antibody (0.2 μg/10 mL) in TBST for 2 h at room temperature, and washed three times with TBST. The blots were incubated for 1 h at room temperature with the secondary antibodies diluted 30,000-fold in TBST, and then washed again three times in TBST. Proteins were visualized by submerging the blots in 100 mM Tris/HCl, 100 mM NaCl, 5 mM MgCl_2_, 0.5 mM Nitro Blue tetrazolium, and 0.5 mM 5-bromo-4-chloroindol-2-yl phosphate (pH 9.5).

## 3. Results

### 3.1. Human Wnt Inhibitory Factor 1 Binds Human Shh with High Affinity

**Figure 1:**
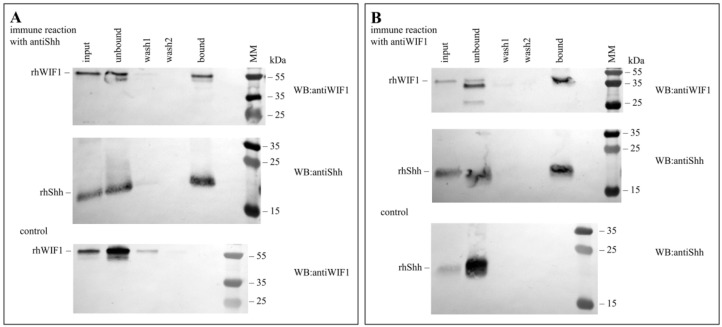
Human WIF1 binds human Shh with high affinity. In pull-down assays, we have incubated rhWIF1 with rhShh, then the solution was mixed with anti-Shh (**A**) or anti-WIF1 antibodies (**B**). The mixtures were pipetted on Pierce Spin Columns filled with Pierce Protein A/G Plus Agarose. After washing, the bound proteins were eluted with Pierce Classic IP kit Elution Buffer (pH 2.8). Samples were analyzed by SDS/PAGE, and the proteins were visualized by Western blotting (WB) with specific antibodies against rhWIF1 and rhShh. In control experiments, we have incubated rhWIF1 with anti-Shh antibody and rhShh with anti-WIF1 antibody. After the affinity binding step samples were visualized by Western blotting with specific antibodies against rhWIF1 and rhShh. Lanes: input, sample applied to the column; unbound fraction; washing fractions, wash1, wash2; bound fraction. The numbers indicate the molecular mass values of proteins of the Page Ruler Plus Protein Ladder (MM).

Pull-down experiments illustrated in Figure 1 have revealed that human WIF1 forms a stable complex with human Shh. 

**Figure 2:**
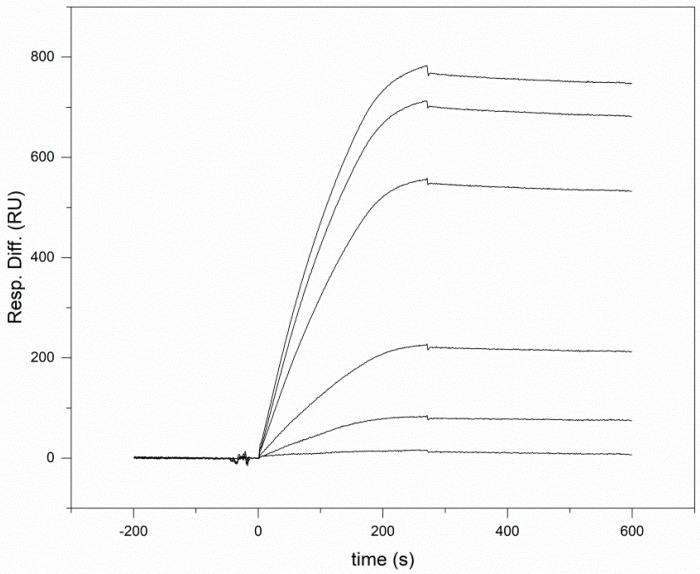
Characterization of the interaction of human sonic hedgehog with human WIF1 by surface plasmon resonance spectroscopy. Various concentrations of rhShh (6.25 nM, 12.5 nM, 25 nM, 50 nM, 75 nM, and 100 nM) were injected over CM5 sensor chips containing immobilized rhWIF1 (at 0 s on the abscissa). For the sake of clarity, the concentrations of the Shh proteins injected over the sensorchips are not indicated; the SPR response increased parallel to the increase in Shh concentration.

In order to obtain quantitative information about the affinity of Shh for WIF1, we have used Surface Plasmon Resonance spectroscopy measurements. When we used immobilized human Wnt Inhibitory Factor 1 as a ligand, injection of human Shh solutions elicited SPR signals in a dose-dependent manner; a representative experiment is shown in Figure 2. Analyses of the SPR response curves of these experiments have revealed that the Shh-WIF1 complex has a K_d_ of 2.06 ± 0.9 nM (Table 1). 

**Figure 3:**
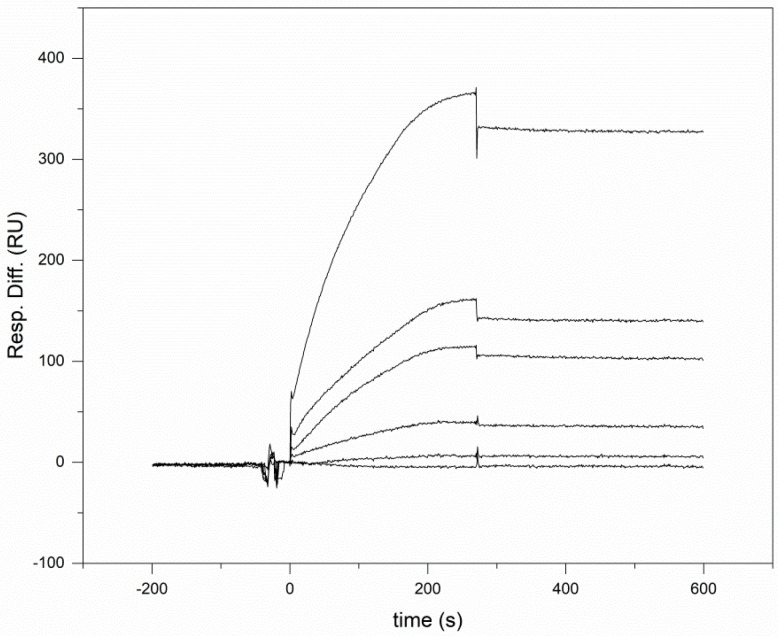
Characterization of the interaction of the WIF domain of WIF1 with recombinant human sonic hedgehog by surface plasmon resonance assays. Various concentrations of rhShh (12.5 nM, 25 nM, 50 nM, 100 nM and 200 nM) were injected over CM5 sensor chips containing immobilized WIF domain (at 0 s on the abscissa). For the sake of clarity, the concentrations of the Shh proteins injected over the sensorchips are not indicated; the SPR response increased parallel to the increase in Shh concentration.

Dose-dependent SPR signals were also observed when we studied the interaction of human Shh with immobilized human WIF domain (Figure 3). Analyses of the SPR response curves (Table 1) have shown that the stability of the Shh-WIF domain complex (K_d_ = 1.18 ± 0.3 nM) is similar to that of the Shh-WIF1 complex, indicating that the WIF domain of WIF1 is primarily responsible for the Shh-WIF1 interaction. 

### 3.2. Human Wnt Inhibitory Factor 1 Is a Potent Antagonist of the Signaling Activity of Human Shh 

In order to assess the biological significance of the interaction of human WIF1 and human Shh, we have used a reporter assay to monitor the effect of WIF1 and its WIF domain on the signaling activity of Shh. The Gli Reporter-NIH3T3 cell line used in this assay contains the firefly luciferase gene under the control of Gli responsive elements stably integrated into NIH3T3 cells. 

**Figure 4:**
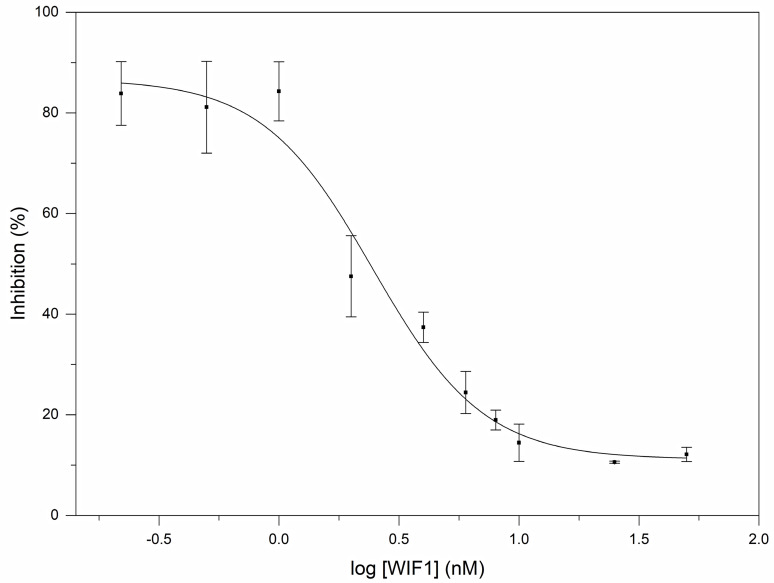
Human WIF1 is a potent inhibitor of the signaling activity of human Shh. In the reporter assays monitoring the hedgehog antagonist activity of WIF1 protein, Gli Reporter-NIH3T3 cells were grown to confluency, and after the removal of the culture medium, the cells were treated with 50 µL aliquots of 0.2 nM rhShh, preincubated for 5 min with 0–50 nM rhWIF1 protein. The luminescence of cells treated with 0.2 nM Shh, 0 nM WIF was 5.88 ± 0.69 fold the luminescence of untreated cells. The figure shows the results of a representative experiment with four parallels. The data are expressed as percentage of the luminescence observed at 0 nM rhWIF1.

Our studies on the influence of human WIF1 on the signaling activity of Shh have revealed that it is a potent antagonist of Shh; a representative experiment is shown in Figure 4. Analyses of the data of reporter assays have shown that human WIF1 protein inhibits the signaling activity of rhShh (0.2 nM) with an EC_50_ value of 2.45 ± 0.032 nM. 

**Figure 5:**
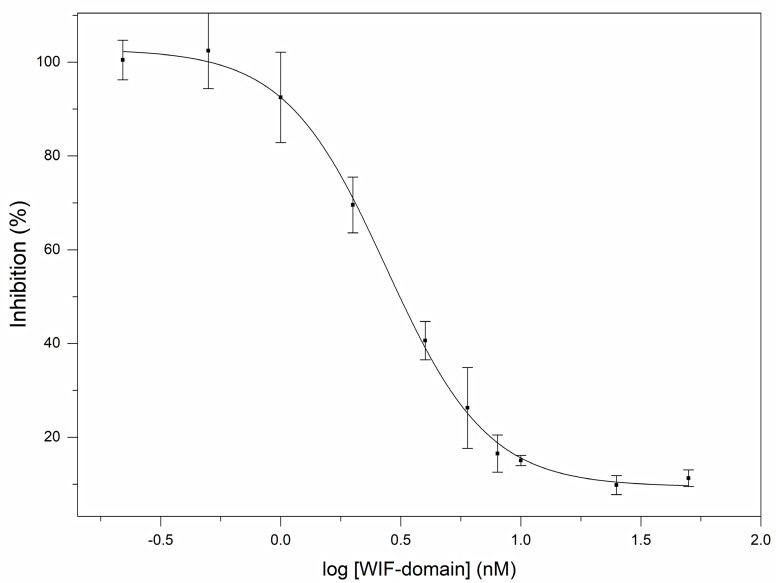
The WIF domain of human WIF1 is a potent inhibitor of the signaling activity of human Shh. In the reporter assays monitoring the hedgehog antagonist activity of the WIF domain of WIF1 protein, Gli Reporter-NIH3T3 cells were grown to confluency, and after the removal of the culture medium, the cells were treated with 50 µL aliquots of 0.2 nM rhShh, preincubated for 5 min with 0–50 nM WIF-domain of WIF1 protein. The luminescence of cells treated with 0.2 nM Shh, 0 nM WIF was 5.88 ± 0.69 fold the luminescence of untreated cells. The figure shows the results of a representative experiment with four parallels. The data are expressed as percentage of the luminescence observed at 0 nM of WIF-domain.

Reporter assays monitoring the influence of the WIF domain on the signaling activity of Shh have shown that it inhibits Shh signaling efficiently; a representative experiment is shown in Figure 5. The WIF domain of the WIF1 protein was found to inhibit the signaling activity of rhShh (0.2 nM) with an EC_50_ value of 2.75 ± 0.135 nM. This value is similar to that for the full-length WIF1 protein, indicating that the WIF domain is primarily responsible for the hedgehog antagonist activity of human WIF1. 

In summary, our Gli reporter assay studies suggest that Wnt inhibitory factor 1 is a potent inhibitor of the signaling activity of sonic hedgehog. 

It must be pointed out, however, that although Gli1 expression is broadly accepted as a marker of hedgehog pathway activation, there are several additional pathways that may also affect the expression and activity of Gli1 (including the Wnt pathway, TGF beta pathway, EGFR pathway, FGFR pathway) [29,30,31,32,33,34,35]. Thus, in principle, the effects of WIF1 on the luminescence of Gli reporter cells might reflect the inhibition of some of the several pathways that may also cause the elevation of Gli1 expression. Our studies, however, exclude this possibility. Neither the WIF1 nor WIF domain had any influence on the luminescence of the unstimulated Gli reporter cells (data not shown). Therefore, the WIF1-sensitivity of the luminescence of the Gli reporter cells is a consequence of the Shh stimulation of cells. Since WIF1 and WIF domain cause the near-complete inhibition of Gli1-activity (Figure 4 and Figure 5), we may conclude that the WIF1 and WIF domain inhibit Gli1–activity elicited by Shh signaling.

## 4. Discussion

In the present work, we have shown that human WIF1 protein binds human Shh with high affinity, and in reporter assays, inhibits its signaling activity efficiently, with a EC_50_ value in the nanomolar range. Our studies have also revealed that the WIF domain of human WIF1 plays a crucial role in the hedgehog antagonist activity of the protein. In this sense, the Wnt antagonist and hedgehog antagonist functions of WIF1 are quite similar in that both functions rely primarily on its WIF domain.

Earlier studies on human WIF1 proteins may provide some insight as to why the WIF domain may interact with both Wnts and hedgehogs. The 3D structure of the recombinant WIF domain of human WIF1 has been determined by NMR spectroscopy [36]. These studies have also shown that a molecule of the detergent Brij-35 is tightly bound through its alkyl chain to an alkyl-binding site of the WIF domain. Since the palmitoleoylation of Wnts is crucial for their activity, we have suggested that this alkyl-binding site serves to bind the lipid moiety of Wnts [36]. Our mutagenesis studies have confirmed that the alkyl-binding site of the WIF-domain plays a significant role in Wnt-binding: the substitution of residues lining this site has a decreased affinity for Wnts [28].

Although the structure of the WIF domain in the complex with Hhs or Wnts have yet to be determined, it seems possible that the lipid-moieties of these morphogens bind to the alkyl-binding site of the WIF domain. According to this hypothesis, the binding of the WIF domain to the lipids shields the very moieties that are crucial for the binding of these morphogens to their cognate receptors. It is noteworthy in this respect that the interaction of Wnt8 with the receptor Frizzled-8 is dominated by the palmitoleic acid side-chain of Wnt8, inserted into the ligand-binding Fz domain of the receptor [37]. Similarly, Shh has been shown to grasp the extracellular domain of its receptor PTCH1 with two lipidic pincers, the N-terminal palmitate and the C-terminal cholesterol, which are both inserted into the PTCH1 protein core [38,39].

Another point that needs discussion is the apparent contradiction of the finding that the binding of human WIF1 to human Shh blocks its activity in reporter assays (present work), whereas in the case of WIF1 of *Drosophila*, its interaction with hedgehog has been found to promote rather than inhibit the activity of this morphogen [23,24].

Numerous examples illustrate that the same growth factor binding protein may serve as either an agonist or antagonist of a given growth factor in a context-dependent, concentration-dependent manner [40,41]. At a high local concentration of growth factor binding proteins (at the site of their synthesis), their inhibitory function may dominate, whereas at lower concentrations (distant from their site of synthesis), they may appear as positive regulators as their stabilizing, transporting functions may be more significant than their inhibitory functions [40,41]. As a recent example, we may cite the case of Scube to illustrate the point that the same morphogen-binding protein may serve both to promote and inhibit the activity of a morphogen. Wierbowski et al. [42] have shown that since Shh is highly hydrophobic, its release and transport from cells requires Scube proteins with which they form soluble complexes; in this context, Scube is a positive regulator of Shh signaling. The soluble Scube-Shh complex, however, cannot signal through the Shh receptor Patched1; in this context, it is a negative regulator of Shh signaling.

Thus, it seems likely that, as a Shh-binding protein, human WIF1 may also function both as an agonist and an antagonist of Shh in a concentration- and context dependent manner. 

Our finding that WIF1 is a potent inhibitor of Shh may call for the reinterpretation of some of the earlier WIF1 loss or gain experiments that have considered WIF1 only as a Wnt-specific inhibitor. A survey of the literature identified two research areas (pathogenesis of anorectal malformations and basal cell carcinomas) that may be relevant in this respect.

During mammalian urorectal development, the urorectal septum descends from the ventral wall of the body to the cloaca membrane to partition the cloaca into urogenital sinus and rectum. Defective urorectal development results in congenital anorectal malformations. Earlier studies on the pathogenetic mechanisms of anorectal malformations revealed that Shh signaling is essential for the development of the distal hindgut: mutant mice with various defects in the Shh signaling pathway exhibit a spectrum of defects mimicking human anorectal malformations [43]. More recent studies, however, revealed that anorectal malformations may also result from the dysregulation of the expression of WIF1 protein [44]. Interestingly, in cultured urorectum addition of the exogenous WIF1 protein induced cloaca membrane disintegration, similar to that observed in Shh^−/−^ mutant embryos [44]. Thus, it seems possible that the effect of WIF1 on urorectal development is exerted, at least in part, through the inhibition of Shh signaling.

The influence of WIF1 on hedgehog signaling in vertebrates may also be relevant for the pathogenesis of basal cell carcinomas. The pivotal defects leading to the formation of these keratinocyte tumors are mutations that result in the aberrant activation of hedgehog signaling [45]. The degree of activation of hedgehog signaling is correlated with the histological appearance: the stronger the activation, the more the tumors resemble basal cell carcinomas [46]. 

It has been shown that the WIF1 protein is secreted by basal cells, accumulates in suprabasal layers, and suppresses proliferation of keratinocytes, cells from which basal cell carcinoma may develop [47]. In view of our observation that WIF1 is a potent inhibitor of Shh, one could speculate that WIF1 secreted by basal cells suppresses the proliferation of keratinocytes by interfering with hedgehog signaling. 

In a recent work, Becker et al. have studied the influence of the forced overexpression of WIF1 on the growth of basal cell carcinomas [48]. The authors have used the cell line ASZ001 that arose as a result of the loss of the functional Ptch1 gene, resulting in the aberrant activation of the hedgehog pathway [49]. WIF1 overexpression was found to inhibit the growth, proliferation, and keratinization of allografts of ASZ001 cells exclusively in vivo, but not in culture, indicating that WIF1-mediated changes require cofactors available in the tumor microenvironment. The authors have demonstrated that the WIF1-mediated inhibition of keratinization and growth of allografts does not involve canonical Wnt signaling. Furthermore, since an overexpression of WIF1 did not affect the expression level of the Gli1 gene, a key target of canonical hedgehog signaling, it has been concluded that “WIF1 does not inhibit hedgehog signaling in mammalian cells” [48]. In our view, the latter conclusion is not justified, since the data do not exclude the involvement of the hedgehog pathway in WIF1-mediated growth inhibition. First, there is evidence that in Ptch1^−/−^ cells, such as ASZ001 cells, Ptch2 mediates the Shh response [50], that like Ptch1, Ptch2 exerts a tumor-suppressive function in basal cell carcinoma cells, and that only after targeting of both paralogs becomes the activation of the hedgehog pathway independent of hedgehog ligand [51]. In other words, despite the Ptch1^−/−^ genotype, the Shh ligand is still perceived by the ASZ001 cell. Second, recent studies have shown that Ptch2 and Ptch1 mediate signaling through distinct modes of Hedgehog ligand reception and divergent signal pathways [52]. The molecular mechanisms leading to the activation of Smo after ligand reception are different in Ptch1/Boc and Ptch2/Gas1-mediated signaling events, resulting in distinct downstream signal pathways. Whereas the transcription of Gli1 in Ptch1-expressing cells increases significantly after Shh addition, in Ptch2-expressing cells, the induction of Gli1 remained at minimal levels. On the other hand, Ptch2-mediated hedgehog signaling induces the significant phosphorylation of Creb and Src proteins, identifying a previously unknown Ptch2-specific signal pathway [52]. Thus, it seems possible that the tumor suppressive effect of WIF1 overexpression on ASZ001 cells may be exerted, at least in part through the inhibition of non-canonical Shh signaling mediated by Ptch2.

Future work is needed to clarify whether the interaction of the human WIF1 protein with hedgehogs has major physiological importance in vertebrates. This question has some medical relevance, since WIF1-based therapies targeting the Wnt pathway may have significant, undesirable side effects if they also affect the Hh pathway. 

## Figures and Tables

**Table 1:** Kinetic parameters of the interaction of human Shh with immobilized human WIF1 protein (WIF1) and with immobilized WIF domain of human WIF1 protein (WIF domain of WIF1). The rate constants of the association and dissociation reactions and the equilibrium dissociation constants of the interactions were determined from surface plasmon resonance measurements with the BIA evaluation software 4.1.

Interacting Proteins	K_d_ (nM)	k_a_ (1/Ms)	k_d_ (1/s)
Shh—WIF1	2.06 ± 0.9	6.85 ± 0.6 × 10^4^	1.41 ± 0.5 ×10^−4^
Shh—WIF domain of WIF1	1.18 ± 0.3	4.26 ± 0.2 × 10^4^	5.03 ± 1.3 × 10^−5^

## Data Availability

The datasets supporting the conclusions of this article are included within the article.
